# The Assessment of Awareness and Attitudes About the Use of Denture Adhesives Amongst Dental Practitioners in Northern Maharashtra: A Delphi Technique

**DOI:** 10.7759/cureus.51833

**Published:** 2024-01-08

**Authors:** Niralee S Bhanushali, Neha B Belsare, Bikash Kumar R Pattanaik, Devanshi G Modi, Pranav P Pund, Prachee R Surwade, Janhavi D Bangar, Sejal V Deore, Lilesh A Shinde

**Affiliations:** 1 Prosthodontics, Mahatma Gandhi Vidyamandir's Karmaveer Bhausaheb Hiray Dental College and Hospital, Nashik, IND

**Keywords:** prosthodontist, post insertion phase, complete denture, delphi technique, denture adhesives

## Abstract

Aim: The goal of this research was to facilitate dialogue and interaction among a group of dental practitioners about their views on denture adhesives, with the objective of reaching a collective consensus through the application of the Delphi Method.

Methods: This investigation employed the Delphi Technique, involving multiple rounds of questionnaires sent to a panel of experts. The objective was to establish a consensus (defined as over 70% agreement) or to explore the different viewpoints on the subject. A comprehensive Delphi questionnaire comprising 22 items was distributed to the Dental Practitioners. The questionnaire covered five key areas concerning denture adhesives: (1) overall perceptions; (2) the role in the development of clinical conditions; (3) specific applications and potential misuses; (4) their importance in denture services, including educating patients about denture adhesives; and (5) a general assessment of their clinical applications.

Results: All 31 panellists, chosen randomly, unanimously agreed to participate, with each of them actively involved in all three rounds of the survey. The panellists reached a consensus and definitively concluded that denture adhesives (1) are beneficial for enhancing the fit and comfort of the prosthesis and are not used to mask the underlying denture problems; (2) cause diseases such as denture stomatitis, candidiasis, and resorption of alveolar bone; (3) have the potential for increasing retention, function, and reducing patient anxiety. However, the panellists did not achieve a consensus on whether denture adhesives cause nausea and gagging in the patients; (4) education is very important for patients with both ill-fitting and well-fitting dentures; (5) are a beneficial adjunct to dentists when fabricating dentures.

Conclusion: The expert panel of distinguished dental practitioners determined that denture adhesives play a valuable supportive role in the field of denture prosthetics, serving important functions during both the creation and post-insertion stages of dentures.

## Introduction

Adhesives were introduced in modern dentistry in the late 15th century. The earliest patent pertaining to adhesive was issued in 1913 and other patents followed this in the 1920s and 1930s. Since then, adhesives have been introduced with different compositions with the aim of providing viscosity and stickiness by absorbing water thus improving the retentiveness of the removable prosthesis [1,2].

The traditional historical position bears a negative attitude toward these products and regards the use of denture adhesives as a poor reflection of their clinical skills and a lack of prosthetic expertise. In contrast, dentists who support the advocate position suggest that denture adhesives can enhance prosthetic denture procedures, patient acceptance, and patient satisfaction [3,4].

The American College of Prosthodontists published evidence-based guidelines regarding the use of denture adhesives and concluded that denture adhesives improve the retention, stability, masticatory function, sealing out of food particles, and overall function of dentures [5-7]. Despite considerable documentation advocating patients’ use of adhesives, many dentists view adhesive usage as a poor reflection of their clinical skills and prosthetic expertise or to provide retention to an ill-fitting prosthesis and even assumed to cause irritation to the denture-bearing tissues in spite of clinical trials failing to prove the same [8]. Many dentists also fear that denture adhesives are causing increased residual ridge resorption (RRR) and soft tissue hyperplasia [1,9,10].

Delphi techniques are used internationally to investigate a wide variety of issues. The Delphi technique is primarily used by researchers when the available knowledge is incomplete or subject to uncertainty and other methods that provide higher levels of evidence cannot be used. The aim is to collect expert-based judgments and often to use them to identify consensus. The aim is to develop an expert-based judgment about an epistemic question. This is based on the assumption that a group of experts and the multitude of associated perspectives will produce a more valid result than a judgment given by an individual expert, even if this expert is the best in his or her field [11].

The conflicting viewpoints about the advantages and disadvantages of denture adhesives among dental professionals have led to slow acceptance of denture adhesives in their practice as a means to enhance denture retention, stability and function. Dentists need to be familiar with denture adhesives to be able to identify those patients who actually need them and to be able to educate them about the advantages, disadvantages and correct use of these products. This is an intriguing topic because it has received so little attention in the formal training of dentists, despite their widespread use among denture wearers [1,6].

The objective of this research was to evaluate the level of awareness and attitudes regarding the use of denture adhesives among Dental practitioners who are actively practising in the North Maharashtra region. This was accomplished through the application of the Delphi technique, a structured method of gathering and refining expert opinions.

## Materials and methods

The items for a Delphi questionnaire are developed by the Delphi users based on the literature on the relevant subject matter in most of the studies investigated [11]. A self-administered survey comprising 22 close-ended questions was distributed to practising Dental practitioners in North Maharashtra using Google Forms. This was done after receiving ethical clearance from the institution's Ethical Committee. The questionnaire had been pre-tested for reliability and validity. Dental practitioners satisfying the inclusion criteria were included in the study. The inclusion criteria were prosthodontists, General practitioners (Bachelor's Degree in Dental Surgery) and Dental Specialists other than prosthodontists, these practitioners should be full-time faculty at an institution and have been in the field of practising dentistry for more than two years and should be from North Maharashtra. The exclusion criteria were the practitioners who were not full-time faculty at an institution, had been in the field of prosthodontists for less than two years and were not from North Maharashtra.

The methodology of this survey was grounded in the Delphi technique, a research approach in social science that seeks to systematically shape and articulate the collective opinion and dialogue of a panel of subject matter experts. This is achieved through successive rounds of questionnaires. The primary aim of employing the Delphi technique is to reach a unified consensus on various questions or, in cases where consensus is not attained, to elucidate the different perspectives and rationales among the panellists for such divergent views.

During round 1, participants selected their responses to the posed questions. Questions that reached a consensus in this first round were considered resolved and thus were not included in the subsequent round. Additionally, in the first-round questionnaire, a section for suggestions regarding language changes was provided. In the second round, only those questions that didn't reach a consensus in the first round were revisited. These were specifically directed at panel members whose views were in the minority for each question. These individuals were then asked to reconsider their stance, deciding whether to align with the majority or continue to hold their minority view. If they chose to maintain their differing opinion, they were required to give a brief rationale for their decision. The third round involved those questions that still lacked consensus after the second round. Here, those in the majority were given the opportunity to formulate counter-arguments to the justifications provided by the minority from the second round, aimed at addressing and possibly resolving the differing viewpoints.

To conduct this Delphi study on denture adhesives, a pre-tested, self-administered questionnaire consisting of 22 close-ended questions containing five domains: (1) overall perceptions; (2) the role in the development of clinical conditions; (3) specific applications and potential misuses; (4) their importance in denture services, including educating patients about denture adhesives; and (5) a general assessment of their clinical applications of denture adhesives was used. The survey responses were organized using a 4-point Likert scale, providing participants with the following options for each question: Strongly Agree, Agree, Disagree, and Strongly Disagree. This scale allowed the panellists to express their level of agreement or disagreement with each statement or question in a clear and quantifiable manner.

The number of Delphi rounds varied relatively widely according to the findings presented in the reviews. The largest range of 0 to 14 rounds was identified by the authors of the review in a medical context. However, the authors did not state the specific research contexts for the extremes of 0 and 14 rounds [11]. The most common number of rounds in the Delphi process was two or three rounds. This study utilized a three-round Delphi process. Following the conclusion of the third round, any questions that failed to achieve consensus were categorized as non-consensus questions. Initially, the round 1 questionnaire, accompanied by a letter briefly introducing the survey, was sent to the panellists. This questionnaire also included questions to gather basic information about the panellists, such as their names, cities, and affiliated institutions. The panellists were asked to return their completed questionnaires within a week. If a panellist did not respond within this timeframe, a phone call reminder was issued, granting them an additional two days to respond. Failure to reply within this extended period resulted in the exclusion of their responses from the data analysis for that specific round.

Round 2 and round 3 were emailed to the panellists with a letter and questions regarding the introduction of the panellist as well. The round 2 questionnaires were emailed to those panellists whose responses were included in the minority group. The round 3 questionnaires were emailed to the panellists that did not achieve consensus after round 2 were presented to those individual panellists of the majority opinion for that given question to develop counter-arguments to the reasons given by those defending minority opinions in round 2 [12].

The analysis of the data was carried out based on these criteria: (1) A consensus opinion was established when there was a minimum agreement of 70%. (2) A majority group was defined as having 51% to 69% agreement on a question. (3) A minority group was identified as having less than 50% agreement. (4) For the primary analysis, the responses from the 4-point scale (Strongly Agree, Agree, Disagree, Strongly Disagree) were consolidated into two main categories (Agree and Disagree) in order to determine the level of consensus for each question in every round.

## Results

Table 1 represents the sociodemographic composition of the panellist group and Table 2 illustrates a list of participating panellists in this study. The panellists were informed to select one choice of answer amongst Strongly Agree (SA), Agree (A), Disagree (D) and Strongly Disagree (SD). The suggestion box which was provided at the bottom of round 1 did not receive any responses, thus there was no change in the language of the questionnaire in round 2 and round 3.

**Table 1:** Sociodemographic Composition of the Panellist Group

Variables	N (%)
Age Group	
21-30	10 (32.25%)
31-40	11 (35.49%)
41-50	6 (19.36%)
>50	4 (12.90%)
Gender	
Male	17 (54.83%)
Female	14 (45.17%)
Qualification	
Prosthodontists	17 (54.83%)
General Practitioners	11 (35.49%)
Other Specialists	3 (9.68%)

**Table 2:** Delphi Technique Panel: A List of 31 Academic Prosthodontists

Panellist	Participating Dental School
Dr. Sanjay Bhavsar	MGV’s KBH Dental College and Hospital, Nashik.
Dr. Girish Nazirkar	SMBT Dental College and Hospital, Sangamner.
Dr. Shailendra Singh	SMBT Dental College and Hospital, Sangamner.
Dr. Umesh Palekar	Rural Dental College, Pravara Institute of Medical Sciences.
Dr. Girija Dodamani	Annasaheb Chudaman Patil Memorial Dental College, Dhule.
Dr. Prabhakar Angadi	SMBT Dental College and Hospital, Sangamner.
Dr. Suresh Nagral	Annasaheb Chudaman Patil Memorial Dental College, Dhule.
Dr. Priyadarshani Pawar	Annasaheb Chudaman Patil Memorial Dental College, Dhule.
Dr. Ashlesha Marathe	SMBT Dental College and Hospital, Sangamner.
Dr. Preetam Mahagaonkar	SMBT Dental College and Hospital, Sangamner.
Dr. Swati Pustake	MGV’s KBH Dental College and Hospital, Nashik.
Dr. Ashish Meshram	MGV’s KBH Dental College and Hospital, Nashik.
Dr. Devyani Shinde	MGV’s KBH Dental College and Hospital, Nashik.
Dr. Damini Thakare	MGV’s KBH Dental College and Hospital, Nashik.
Dr. Akshay Bhandari	MGV’s KBH Dental College and Hospital, Nashik.
Dr. Mohit Patil	SMBT Dental College and Hospital, Sangamner.
Dr. Sneha Tarle	MGV’s KBH Dental College and Hospital, Nashik.
Dr. Sonali Khillare	Seth Nandlal Dhoot Hospital Aurangabad.
Dr.Vishakha Tagade	MGV’s KBH Dental College and Hospital, Nashik.
Dr. Rutuja Sanap	SMBT Dental College and Hospital, Sangamner.
Dr. Ashwini Sonawane	SMBT Institute of Dental Sciences and Research, Igatpuri.
Dr. Swarali Shelke	R. R. Kambe Dental College and Hospital, Akola.
Dr. Abhishek Bezalwar	SMBT Institute of Dental Sciences and Research, Igatpuri.
Dr. Abhishek Kurdukar	SMBT Institute of Dental Sciences and Research, Igatpuri.
Dr. Snehal Markandey	VYWS Dental College and Hospital Amravati.
Dr. Shreyas S Ingle	Ranjeet Deshmukh Dental College & Research Center, Nagpur.
Dr. Saima Tabassum	R. R. Kambe Dental College and Hospital, Akola.
Dr. Pratik Gupta	R. R. Kambe Dental College and Hospital, Akola.
Dr. Chetan Palaskar	Ranjeet Deshmukh Dental College & Research Center, Nagpur.
Dr. Vinay Sharma	SMBT Institute of Dental Sciences and Research, Igatpuri.
Dr. Pawan Patel	SMBT Institute of Dental Sciences and Research, Igatpuri.

In response to all the sub-questions that addressed the attitude of dentists regarding the use of denture adhesives in denture patients. Panellists agreed that denture adhesives enhance the fit of the prosthesis (100%) and provide psychological comfort to denture patients (96.7%). Panellists disagreed that denture adhesives are used by dentists to mask underlying denture problems (72.7%) (Table 3).

**Table 3:** Topic I: Attitude of Dentists Regarding the Use of Denture Adhesives in Denture Patients

Sr. no	Question	SA	A	D	SD
1.	Enhancing the fit of the prosthesis (i.e., the retention and stability).	19.4.%	80.6%	0%	0%
	The panel achieved a consensus with 100% agreeing on round 1 for this question.				
2.	Providing psychological comfort to the denture patient.	29%	67.7%	3.3%	0%
	The panel achieved a consensus with 96.7% agreeing on round 1 for this question.				
3.	Masking underlying denture problems.	9.8%	17.5%	55.5%	17.2%
	The panel achieved a consensus with 72.7% disagreeing on round 1 for this question.				

The panellists agreed that denture adhesives cause the development of clinical conditions such as candidiasis (77.5%) and disagreed that denture adhesives are responsible for the development of oral cancer (77.4%) and leukoplakia (71.6%) in round 1 (Table 4). The question regarding the development of denture stomatitis received 57.2% agreement and 42.8% disagreement in round 1 and later received a consensus of 85% agreement in round 2. The panellists who wanted to maintain their minority position were given a box to justify the reason for doing the same. Panellists stated that denture adhesives do not cause denture stomatitis as it depends on the oral hygiene of the patient, underlying clinical conditions, duration of usage of denture adhesives, and clinical correctness of the prosthesis and it is extremely rare. In the same way, the question which stated that denture adhesives contribute to the deterioration of the alveolar ridge due to irritation of the surrounding tissues received 67.8% agreement and 32.2% disagreement in round 1 and later received 85% agreement in round 2 (Table 4). The panellists who wanted to maintain their minority position stated the reasons that resorption of alveolar bone is dependent on the improper use of denture by the patient such as not wearing the denture daily or not removing the denture at night.

**Table 4:** Topic II: Development of Clinical Conditions Related to the Use of Denture Adhesives

Sr. no.	Question	SA	A	D	SD
4.	Oral Cancer	3.2%	19.4%	67.7%	9.7%
	The panel achieved a consensus with 77.4% disagreeing on round 1 for this question.				
5.	Denture Stomatitis	25%	60%	15%	0%
	The panel achieved a consensus with 85% agreeing on round 2 for this question.				
6.	Leukoplakia	6.5%	21.9%	54.9%	16.7%
	The panel achieved a consensus with 71.6% disagreeing on round 1 for this question.				
7.	Candidiasis	19.4%	58.1%	19.4%	3.1%
	The panel achieved a consensus with 77.5% agreeing on round 1 for this question.				
8.	Resorption of the alveolar bone as a result of tissue irritation	20%	65%	15%	0%
	The panel achieved a consensus with 85% agreeing on round 2 for this question.				

The panellists agreed that denture adhesives are used to stabilize trial bases during denture fabrication (93.6%), to prevent patients' fear during try-in (93.6%), to ensure proper function and comfort to the patient (90.4%), to overcome patients anxiety after denture insertion (87.1%), to provide retention and stability in patients with poor oral anatomy (96.8%) in round 1. The panellists agreed that denture adhesives are most effective in loose-fitting mandibular complete dentures (94.2%) in round 1 (Table 5).

**Table 5:** Topic III: Specific Clinical Applications and Potential Misuses of Denture Adhesives

Sr. no	Question	SA	A	D	SD
9.	To stabilize trial bases in the early stages of denture fabrication (i.e., while recording centric and vertical relation)	35.5%	51.6%	9.7%	3.2%
	The panel achieved a consensus with 93.6% agreeing on round 1 for this question.				
10.	To allay the patient’s fears at the trial arrangement of teeth on denture bases visit	35.5%	58.1%	6.5%	0%
	The panel achieved a consensus with 93.6% agreeing on round 1 for this question.				
11.	To augment retention, comfort, and function during the interim period after insertion of immediate dentures	19.4%	71%	9.7%	0%
	The panel achieved a consensus with 90.4% agreeing on round 1 for this question.				
12.	To overcome patients’ anxiety for a short period (2-3 weeks) after insertion of new complete dentures (ie, not immediate dentures)	19.4%	67.7%	12.9%	0%
	The panel achieved a consensus with 87.1% agreeing on round 1 for this question.				
13.	To provide additional retention and stability for patients who have inadequate oral anatomy	19.4%	77.4%	0%	3.2%
	The panel achieved a consensus with 96.8% agreeing on round 1 for this question.				
14.	Patients with poor oral hygiene maintenance should not use Denture Adhesives	22.6%	67.7%	9.7%	0%
	The panel achieved a consensus with 90.3% agreeing on round 1 for this question.				
15.	Prescribing Denture Adhesives indicates inadequate skills of the Clinician to Fabricate Dentures	0%	19.4%	58.1%	22.6%
	The panel achieved a consensus with 80.7% disagreeing on round 1 for this question.				
16.	Use of Denture Adhesives is more effective with respect to loose-fitting Maxillary Complete Denture	20%	70%	5%	5%
	The panel achieved a consensus with 90% agreeing on round 2 for this question.				
17.	Use of Denture Adhesives is more effective with respect to loose fitting Mandibular Complete Denture	6.5%	67.7%	22.6%	3.2%
	The panel achieved a consensus with 74.2% agreeing on round 1 for this question.				
18.	Denture Adhesives cause Nausea to most of the patients	0%	50%	45%	5%
	The panel did not achieve a consensus on round 3 with 50% agreeing and 45% disagreeing for this question.				
19.	Denture Adhesives cause Gagging to most of the patients	0%	45%	50%	5%
	The panel did not achieve a consensus on round 3 with 45% agreeing and 50% disagreeing for this question.				

The question regarding whether the denture adhesives are most effective in loose-fitting maxillary complete dentures received 67.8% agreement and 32.3% disagreement in round 1. The reasons given by panellists to remain in the minority position were that denture adhesives are effective for loose-fitting maxillary complete dentures but most panellists suggested that the effectiveness of denture adhesives should be more for loose-fitting mandibular dentures as mostly the mandibular ridge is more resorbed in comparison with the maxillary ridge. The panellists disagreed that the use of denture adhesives resembles the lack of clinical skills of the practitioner (80.7%) in round 1.

Regarding question 18 which received 50% agreement and 45% disagreement in round 3 and thus failed to achieve consensus. Panellists who maintained the minority position stated reasons that denture adhesives do not cause nausea in most of the patients and it is quite rare. Question 19 received 45% agreement and 50% disagreement in round 3 and failed to achieve consensus. Panellists who maintained the minority position stated reasons that denture adhesives are soggy and sticky in nature and thus might cause psychological gagging to the patient (Table 5).

The panellists concurred that educating patients about the use of denture adhesives is a crucial aspect of care for both those with poorly fitting dentures (93.6%) and those with well-fitting dentures (80.6%), as seen in the first round (Table 6). Additionally, they agreed that denture adhesives are a valuable aid for dentists during the fabrication of dentures, with an (90.3%) agreement in the first round (Table 7).

**Table 6:** Topic IV: Patient Education

Sr. no	Question	SA	A	D	SD
20.	Patient education on the use (appropriate and/or inappropriate) of denture adhesives is an important part of denture service for patients with ill-fitting denture	48.4%	45.2%	6.5%	0%
	The panel achieved a consensus with 93.6% agreeing on round 1 for this question.				
21.	Patient education on the use (appropriate and/or inappropriate) of denture adhesives is an important part of denture service for patients with well-fitting denture	25.8%	54.8%	19.4	0%
	The panel achieved a consensus with 80.6% agreeing on round 1 for this question.				

**Table 7:** Topic V: Overall Opinion of Denture Adhesives

Sr. no	Question	SA	A	D	SD
22.	Overall, denture adhesives can be a beneficial adjunct to the dentist when fabricating dentures	29%	61.3%	9.7%	0%
	The panel achieved a consensus with 90.3% agreeing on round 1 for this question.				

## Discussion

The panel demonstrated strong engagement and collaboration, as evidenced by a 100% response rate across all three rounds of the questionnaire, along with their promptness in returning each round of the survey. The diverse perspectives of the panellists contributed to a clear understanding of dentists' awareness and attitudes toward the use of denture adhesives.

The survey data revealed that in round 1, only 5 out of 22 questions (representing 77.2% of the total) did not reach consensus. In round 2, consensus was achieved on three of these questions, bringing the consensus rate to 90%. By round 3, consensus was reached on all but 2 of the 22 questions, also amounting to a 90% consensus rate. Therefore, the majority of the questions quickly reached a consensus. The survey included questions categorized under five main topics related to denture adhesive usage.

Topic I: Attitude of dentists regarding the use of denture adhesives in denture patients

Panellists achieved rapid consensus on round 1 on all three questions. They agreed that denture adhesives enhance the fit of the prosthesis and provide psychological comfort to patients. Ann Slaughter et al. [12] reported similar results where panellists agreed to these questions rapidly. The panellists agreed that denture adhesives are not used to mask underlying denture problems. This finding contradicted the findings from the study by Deshmukh M et al. [13], where the dentists agreed that denture adhesives are used to mask denture fabrication and processing errors.

Topic II: Development of clinical conditions related to the use of denture adhesives

The panellists agreed that denture adhesives cause the development of clinical conditions such as candidiasis, and denture stomatitis but not oral cancer and leukoplakia. This finding coincided with the findings from a previous study where denture adhesives develop denture stomatitis and an imbalance in the oral flora due to microbial contamination and candidiasis because of denture adhesive, more than 65% of the dentists agreed [14]. Panellists agreed that denture adhesives cause resorption of alveolar bone as a result of irritation. Slaughter A et al. [12] reported the same result.

Topic III: Specific clinical applications and potential misuses of denture adhesives

The panellists agreed that denture adhesives are used to stabilize trial bases, allay the patients’ fears augment retention, comfort, and function during the interim period after insertion of immediate dentures, overcome patients’ anxiety, provide additional retention and stability for patients who have inadequate oral anatomy and should not be used for patients with poor oral hygiene. This was consistent with the consensus reached by the dental practitioners in the study by Mantri et al. [1] where the reason being stable record bases is a prerequisite for recording accurate jaw relations. Stafford et al. [15] indicated that denture adhesives could influence oral flora by causing an imbalance in the flora and thus could lead to poorer oral health. The panellists disagreed that the use of denture adhesives indicates poor clinical skills of the practitioner. This result coincided with Thakur SN et al. [5]. The panellists did not achieve consensus on the question that denture adhesives cause nausea and vomiting in the patient.

Topic IV: Patient education

The panellists agreed that the knowledge of the use of denture adhesives is very crucial for patients with well-fitting dentures or ill-fitting dentures. This coincided with the findings in Slaughter A et al. [12]. Knowledge regarding the proper use of denture adhesives should be given to the patients such as cleaning the old denture adhesive before applying new, using sparingly, cleaning the tissues well before applying denture adhesives, and discontinuing if it causes any problems.

Topic V: Overall opinion of denture adhesives

The outcomes of this research demonstrate that denture adhesives serve as a useful aid for Dental practitioners in the creation of dentures.

A primary limitation of this study is the limited number of participants, which constrains the scope of the findings and their applicability beyond the specific region studied. Future research should aim to bridge this gap by encompassing a broader geographical area and including a larger pool of dentists.

## Conclusions

The panel reached a consensus on various aspects of denture adhesive use, agreeing that these adhesives enhance the fit of prostheses, provide psychological comfort to patients, and are not used to mask problems with the underlying denture. It was also acknowledged that while denture adhesives can contribute to conditions like candidiasis, denture stomatitis, and alveolar ridge resorption, they are not associated with oral cancer or leukoplakia. The panellists noted that these adhesives aid in stabilizing trial bases during the fabrication of dentures, reducing patient anxiety during trials, and ensuring comfort and proper function after insertion. They are particularly beneficial for improving retention and stability in patients with poor oral anatomy and are most effective in loose-fitting upper and lower complete dentures. However, there was no consensus on whether denture adhesives cause nausea and gagging. The importance of educating patients about the use of adhesives for both ill-fitting and well-fitting dentures was emphasized, highlighting their value as a tool for dentists in denture fabrication.
